# Microbial detoxification of eleven food and feed contaminating trichothecene mycotoxins

**DOI:** 10.1186/s12896-017-0352-7

**Published:** 2017-03-15

**Authors:** Rafiq Ahad, Ting Zhou, Dion Lepp, K. Peter Pauls

**Affiliations:** 1 Guelph Food Research Centre, Agriculture and Agri-Food Canada, 93 Stone Road West, Guelph, Ontario N1G 5C9 Canada; 2 Department of Plant Agriculture, University of Guelph, Guelph, Ontario N1G 2W1 Canada

**Keywords:** Biodetoxification, Food contamination, *Fusarium* mycotoxins, Microbial de-epoxidation, Trichothecenes

## Abstract

**Background:**

Contamination of agricultural commodities with multiple trichothecene mycotoxins, produced by toxigenic *Fusarium* species, is a food safety issue, which greatly affects grain production and marketing worldwide. Importantly, exposure to multiple trichothecenes may increase toxicity in animals due to their synergistic and/or additive effects. To address the problem this study aimed to achieve a novel biological trait capable of detoxifying various food and feed contaminating trichothecenes under aerobic and anaerobic conditions and wide range of temperatures.

**Results:**

A highly enriched microbial consortium (called DX100) capable of transforming eleven trichothecenes to significantly less toxic de-epoxy forms was achieved after prolonged incubation of soil microbial culture with 200 μg/mL deoxynivalenol (DON). DX100 demonstrated de-epoxidation activity under aerobic and anaerobic conditions, a greater range of temperatures and around neutral pH. The consortium contains 70% known and 30% unknown bacterial species, dominated by *Stenotrophomonas* species. Probably novel bacteria including strains of *Stenotrophomonas* and *Alkaliphilus*-*Blautia* species complex could be involved in aerobic and anaerobic de-epoxidation of trichothecenes, respectively. DX100 showed rapid and stable activity by de-epoxidizing 100% of 50 μg/mL deoxynivalenol at 48 h of incubation and retaining de-epoxidation ability after 100 subcultures in mineral salts broth (MSB). It was able to de-epoxidize high concentration of DON (500 μg/mL), and transformed ten more food contaminating trichothecenes into de-epoxy forms and/or other known/unknown compounds. Microbial de-epoxidation rate increased with increasing trichothecene concentrations in the broth media, suggesting that DX100 maintains a robust trichothecene detoxifying mechanism. Furthermore, the nature of microbial de-epoxidation reaction and inhibition of the reaction by sodium azide and the finding that bacterial cell culture lysate retained activity suggests that certain cytoplasmic reductases may be responsible for the de-epoxidation activity.

**Conclusions:**

This study reports the enrichment procedure for obtaining an effective and stable microbial consortium DX100 capable of de-epoxidizing several food contaminating trichothecene mycotoxins. DX100, which has de-epoxidation ability under wide range of conditions, represents a unique enzymatic source which has great industrial potential for reducing contamination of foods/feeds with multiple trichothecenes, and minimizing their synergistic/additive cytotoxic effects on consumer health.

## Background

The toxigenic *Fusarium* (mold) and other fungal pathogens contaminate cereal-based foods and feeds by producing a variety of health injurious trichothecene mycotoxins [1]. The pathogens deposit multiple mycotoxins in grain during the course of disease development in the crop field and during post-harvest storage [2, 3]. Importantly, the fungal toxins often enter the food chain [4], and exposures to them through consumption may cause serious mycotoxicosis in humans and animals [5–8].

Trichothecenes are chemically tricyclic sesquiterpenes, characterized by a double bond at the C9, 10 position and a C12, 13 epoxy functional group. The latter is the principal moiety responsible for the toxicity of these fungal secondary metabolites in eukaryotic organisms [9]. Type-A and B are the two types of fungal trichothecenes that often co-contaminate cereals [1, 10]. The two types of trichothecenes are separated by the presence of a ketone at position C8 in type-B [(e.g. DON (deoxynivalenol), also known as vomitoxin], which is absent in type-A toxins (e.g. T2-toxin, HT-2 toxin) [11]. Although the toxicities of the members of two trichothecene types vary, exposure to multiple mycotoxins may increase their cytotoxicity because of their synergistic and/or additive cytotoxic effects on eukaryotic organisms [12, 13]. Common toxicity effects of the trichothecenes in humans and animals include diarrhea, vomiting, feed refusal, growth retardation, immunosuppression, reduced ovarian functions/reproductive disorders and even death [14, 15].

The magnitude of food contamination with trichothecenes is influenced by various biotic and abiotic factors, and the risk can be further increased due to climate change [16]. Therefore, reduction of the toxins levels in contaminated foods remains a very difficult task [17]. Physical and chemical processes to remove trichothecenes from foods and feeds are either detrimental, ineffective or prohibitively expensive [18–20]. However, the use of detoxifying microorganisms to biodegrade mycotoxins, through de-epoxidation or other mechanisms, could be an effective alternative [21, 22]. Previous studies suggested that the toxicity of DON is mainly due to the epoxide moiety, and there is a significant reduction of DON toxicity (by 54%) when it is reduced to produce de-epoxy DON [23, 24]. These studies further revealed reductions of nivalenol (NIV) and T-2 toxin toxicities by 55 and 400%, respectively, when their de-epoxidized derivatives were tested.

The potential value of microorganisms with trichothecene de-epoxidation activity for improving food/feed quality and safety has led to the identification of bacteria isolated from chicken intestine and rumen fluid, that are able to cleave the epoxide ring of food contaminating trichothecenes under anaerobic conditions and relatively high temperature (~37 °C) [25, 26]. Furthermore, the anaerobic bacterial strain BBSH 797, which can de-epoxidize DON, scirpentriol and T-2 triol trichothecene mycotoxins, has been used as a feed additive for poultry and swine diets [27, 28]. Earlier attempts were made to obtain de-epoxidizing aerobic bacteria, but the culture did not retain DON/NIV de-epoxidation activity after subculturing [29, 30].

In the process of developing effective biotechnological strategies for eliminating trichothecenes from contaminated agricultural commodities, we previously reported an aerobic microbial consortium with a low rate of DON de-epoxidation activity, most likely due to the existence of undesired fast growing microorganisms in the culture [31]. The present study aimed to increase its de-epoxidation activity through selective enrichment of that culture, and to characterize the enriched culture including its ability to de-epoxidize a variety of *Fusarium* produced trichothecenes under aerobic and anaerobic conditions and wide range of temperatures.

## Methods

### Media and chemicals used

The media used in this study were: minimal medium (MM) = six mineral salts and 10.0 g/L sucrose [31], MSB = six mineral salts containing 0.5% Bacto Peptone [31], MSC = MSB containing reduced quantity (0.3%) Bacto Peptone, MSY = six mineral salts + 0.5% yeast extract, MS-DON = six mineral salts + 200 μg/mL DON, SE = sterile water-soluble soil extract [31], PDB = potato dextrose broth, LB = Luria Bertani, NB = nutrient broth and 1/10 NB = one-tenth strength NB. The pH of the media was adjusted as needed before autoclaving. Solid media were prepared by adding 1.5% agar. The media ingredients and prepared media were purchased from Sigma-Aldrich (Oakville, ON, Canada), Fisher Scientific (Fair Lawn, New Jersey, USA), and/or Difco (Sparks, Maryland, USA). All trichothecene mycotoxins were obtained from Biomin (Romer Lab, Union, MO, USA).

### Enrichment and functional stability of trichothecene de-epoxidizing microorganisms

A soil microbial culture having low level of DON de-epoxidation activity originating from southern Ontario, Canada was used for enrichment [31]. An aliquot (20 μL) of the culture was transferred to a 1.5 mL eppendorf tube containing 0.5 mL MSB (pH 7.0). The culture was spiked with 200 μg/mL pure DON and incubated with continuous shaking at 220 revolutions per minute (rpm) at room temperature. After 7–10 days of incubation, an aliquot (10 μL) of the culture was transferred to fresh MSB containing 200 μg/mL DON. In negative controls no DX100 was added. This procedure was repeated twelve times and the microbial consortium was designated DX100. Before every transfer to fresh MSB the de-epoxidation activity of the culture was examined by using a standard method, liquid chromatography-ultraviolet-mass spectrometry (LC-UV-MS) [32]. Briefly, equal volumes of the bacterial culture and methanol were mixed and the samples were incubated for 30 min at room temperature to lyse the bacterial cells. Insoluble cell materials and media components in the mixture were eliminated by passing it through a 0.45 μm polyvinylidene difluoride membrane filter (Whatman®, Florham Park, New Jersey, USA). The percentage of DON to dE-DON (de-epoxy deoxynivalenol), also known as DOM-1, transformation in the filtrate was determined by LC-UV-MS technique [32].

To determine the stability of the microbial de-epoxidation activity an active culture was subcultured for one hundred generations in MSB containing 50 μg/mL DON. The final subculture was tested for its de-epoxidation activity using LC-UV-MS technique.

### 16S rRNA metagenomic library preparation and sequencing

Genomic DNA was extracted from the highly enriched bacterial culture using Gentra Puregene Yeast/Bacteria Kit (Qiagen Inc., Mississauga, Ontario, Canada). Amplicon libraries of the V3-4 region of the 16S rRNA gene were prepared following the procedure of Illumina 16S Metagenomic Sequencing Library Preparation Guide (Rev. B). Briefly, a ~ 550 bp fragment of the 16S rRNA V3-4 region was amplified using the primers Bakt 341F (5-cctacgggnggcwgcag) and Bakt 805R (5-gactachvgggtatctaatcc) [33]. The Bakt 341F and Bakt 805R primers contained 5-tcgtcggcagcgtcagatgtgtataagagacag and 5-gtctcgtgggctcggagatgtgtataagagacag 5’ Illumina overhang adapter sequences, respectively. Each 25 μl volume reaction mixture contained 12.5 ng of template DNA, 200 nM of each primer and 1x KAPA HiFi HotStart ReadyMix (VWR, Mississauga, ON, Canada). PCR was performed following the conditions: 95 °C (3 min), 25 cycles of 95 °C (30 s), 55 °C (30 s) and 72 °C (30 s), followed by 72 °C (5 min). The PCR amplicons were purified using Ampure XP beads (Beckman Coulter, Mississauga, ON, Canada). Sequencing adapters containing 8 bp indices were incorporated into the 3’ and 5’ ends of the purified amplicons by PCR using the Nextera XT Index kit (Illumina, San Diego, CA, USA). The 50 μl PCR reaction contained 5 μl PCR amplicon, 5 μl each indexing primer, and 25 μl 2x KAPA HiFi HotStart ReadyMix. PCR was performed under the following conditions: 95 °C (3 min), 8 cycles of 95 °C (30 s), 55 °C (30 s) and 72 °C (30 s), followed by 72 °C (5 min). The PCR amplicons were purified with Ampure XP bead and quantified using the Quant-iT PicoGreen double-stranded DNA assay (Invitrogen/Life Technologies Inc., Burlington, ON, Canada). After pooling equimolar amounts of amplicons, 5% equimolar of PhiX DNA (Illumina) was added, and sequencing was performed on an Illumina MiSeq instrument, using the Illumina MiSeq 600-cycle v3 kit. Taxonomic composition of the sequences was determined with Illumina MiSeq Reporter v.2.5.1 using the metagenomics workflow.

### Transformation of A- and B type trichothecene mycotoxins

DX100 was examined for its ability to degrade five type-A trichothecene mycotoxins (diacetoxyscirpenol, HT-2 toxin, neosolaniol, T-2 toxin and T2-triol) and five type-B trichothecene mycotoxins (3-acetyl-deoxynivalenol, 15-acetyl-deoxynivalenol, fusarenonX, nivalenol and verrucarol). One hundred and fifty micrograms of each mycotoxin were added to 1.5 mL NB in 5 mL microcentrifuge tubes (Sigma Aldrich), resulting in a 100 μg/mL mycotoxin solutions. Each tube was inoculated with 100 μL of an overnight DX100 culture, and the cultures in NB + mycotoxin were incubated at 28 °C with continuous shaking at 180 rpm, while the negative controls lacked DX100. The percentage of trichothecene transformation to different compounds (including de-epoxy and de-acetylated forms) by the culture was analyzed [32]. Five replicate cultures were evaluated for each trichothecene mycotoxin.

### Effects of DON concentration, substrate, temperature, pH, and aeration on microbial de-epoxidation activity

To determine the effects of DON concentration on the growth and de-epoxidation activity of DX100, tubes containing 3 mL of NB or MSB spiked with low (20 μg/mL), high (200 μg/mL) or very high (500 μg/mL) levels of DON were inoculated with DX100. The cultures were grown for 16 h with continuous shaking at 220 rpm and then the cell density was adjusted to 0.25 OD_600nm_ (10^7^ cells/mL). While incubating the culture at 28 °C with shaking at 180 rpm, 1.5 ml was removed every 12 h and the OD_600nm_ was measured. The same volume of microbial culture (bacteria and broth) was analyzed for de-epoxidation activity by LC-UV-MS technique [32]. Three replicate cultures for each mycotoxin were tested.

The effect of oxygen was evaluated by inoculating 5 mL of NB or MSB in 15 mL falcon tubes with an overnight culture of DX100. A moderate amount of DON (50 μg/mL) was added to the cultures and they were incubated at 28 °C with shaking at 180 rpm. Bacterial growth (OD_600nm_) and de-epoxidation activity were determined every 6 h [32]. The assay was repeated with MSB medium under anaerobic conditions [31]. Three replicate cultures were considered for each experiment.

To examine the effects of substrate on microbial de-epoxidation activity DX100 was grown at 28 °C for 72 h in NB, 1/10 NB, MM, MS-DON, MSB, MSC, MSY, SE, PDB or LB containing 50 μg/mL DON. The media pH values for this assay were maintained at pH 7.0 ± 0.2. To evaluate the effect of temperature, MSB containing 50 μg/mL DON was inoculated with DX100 and incubated at 10, 15, 20, 25, 30, 35, 40, 45 or 50 °C. The effect of pH on growth and de-epoxidation of DX100 was studied with MSB + 50 μg/mL DON adjusted to pH 5.5, 6.0, 6.5, 7.0, 7.5 or 8.0, and the cultures were grown at 28 °C for 72 h. Three replicate cultures were tested for each experiment.

### Effects of bacterial energy inhibitor, and localization of microbial de-epoxidation enzyme

In order to determine if the trichothecene de-epoxidation is bacterial and enzymatic, overnight cultures of DX100 grown in MSB containing 50 μg/mL DON were treated with 0.01% and 0.001% (w/v) of the bacterial energy inhibitor, sodium azide (NaN_3_) (Sigma-Aldrich), which blocks gram-negative bacterial electron transport [34]. Controls were prepared without SA, and three replicate cultures were maintained for each treatment. The de-epoxidation activity of the DX100 culture was analyzed after incubation at 28 °C for 72 h with shaking at 180 rpm [32].

In a separate assay, bacterial cell lysates were prepared using B-PER II Bacterial Protein Extraction Reagent following the manufacturer’s instruction (PI 78260, Thermo Scientific, Rockford, IL, USA). DX100 was cultured for 48 h in 1.5 mL MSB under the conditions described above. After collecting the cell pellet by centrifuging one-third (0.5 mL) of DX100 culture at 5000 × g for 10 min 2 mL B-PER II reagent was added to it. The cells were disrupted by pipetting the pellet suspension up and down several times. Immediately 20 μL Halt Protease Inhibitor Cocktail (Thermo Scientific, Rockford, IL, USA) was added to the cell lysate. To prepare culture filtrate the DX100 culture (0.5 mL) was passed through a 0.22 μm EMD filter Millex^TM^ Sterile Syringe Filters: Durapore^TM^ PVFD Membrane (Fisher Scientific). The remaining 0.5 mL of the DX100 culture was used as positive control. Cell lysate, culture filtrate and control were spiked with 50 μg/mL DON and incubated at 28 °C. The de-epoxidation activity of the cell lysate, culture filtrate and control were analyzed after a 72 h incubation [32]. Three replicate cultures were tested.

### Data analysis

Data were organized using Microsoft Excel Program 2007, and the statistical analyses were carried out using SAS procedures of General Linear Model for Windows version 9.1 (SAS Institute, Cary, NC, USA, 2002–03). Average values of the replications were expressed as means ± standard errors. The mean differences among treatments were considered significant at *p* < 0.05 according to Tukey’s-HSD test.

## Results

### Microbial enrichment, functional stability and identification of bacteria in trichothecene transforming bacterial consortium

**Fig. 1:**
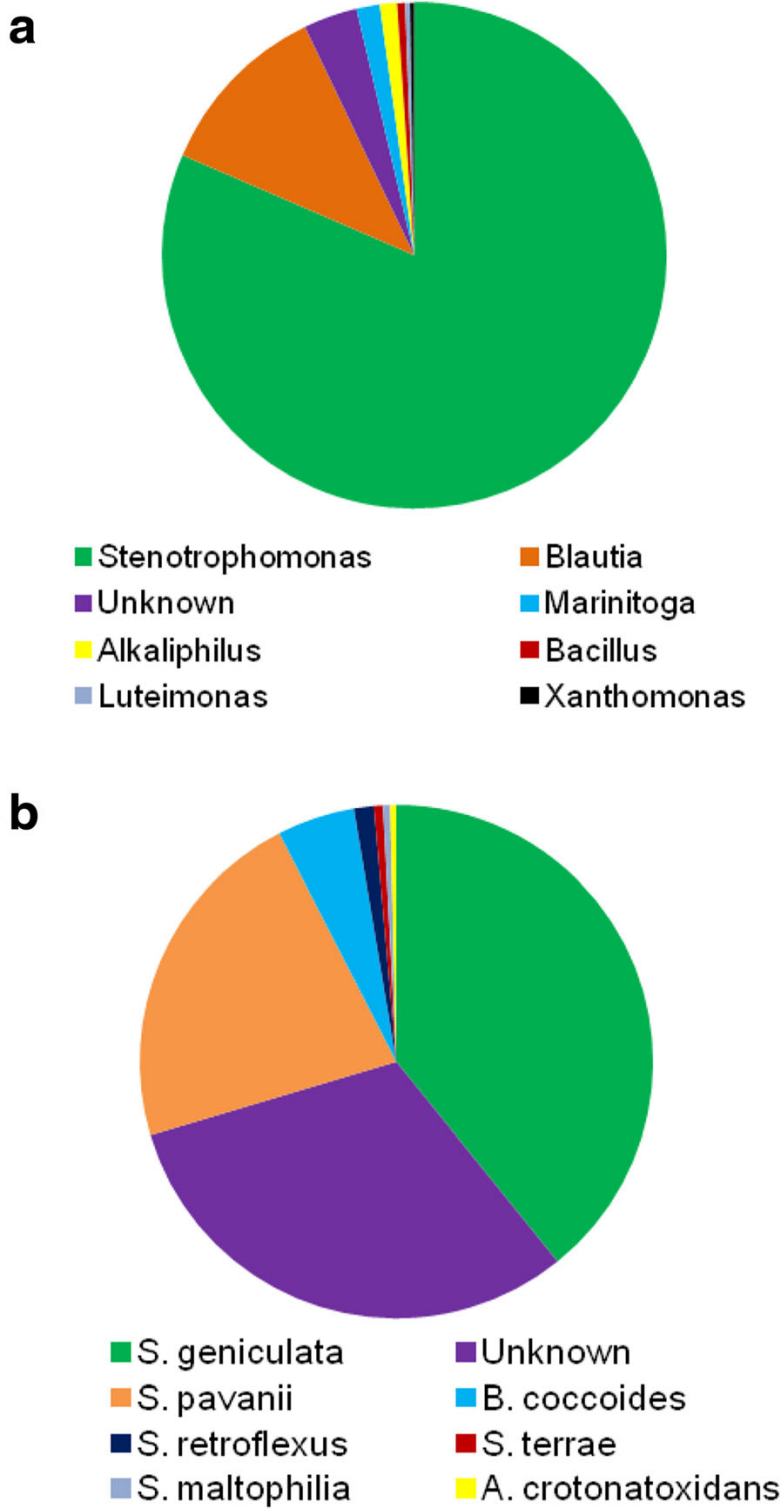
Identification and abundance of bacterial genera and species in trichothecene de-epoxidizing DX100 microbial consortium. **a**: Relative abundance of known and unknown bacterial genera in DX100. **b**: Relative abundance of known and unknown bacterial species in DX100. The areas of different color represent abundance of different bacterial genera or species present in DX100. The initials of bacterial species A, B and S indicate genera *Alkaliphilus*, *Blautia* and *Stenotrophomonas*, respectively

Three months of co-incubating the soil microbes with 200 μg/mL DON in MSB led to an enriched bacterial consortium DX100 that efficiently de-epoxidized trichothecenes, after 100 subcultures in broth. The next generation metagenomic sequencing technology determined an altered composition of the bacterial consortium in DX100 compared to the original culture that was used for enrichment. The enriched DX100 consortium is comprised of different known and unknown bacterial genera, dominated by aerobic *Stenotrophomonas* (80.03%), followed by anaerobic *Blautia* (11.19%) and unknown microbes (3.38%) (Fig. 1a). In contrast, the initial culture contained a heterogenous community comprised of species of aerobic and anaerobic bacterial genera (namely, *Serratia, Clostridium, Citrobacter, Enterococcus, Stenotrophomonas and Streptomyces*), and dominated by *Serratia* [35]. Further bioinformatics analyses elucidated the percentage occurrences of known and unknown bacterial species in DX100 (Fig. 1b). The predominant species was identified to be *Stenotrophomonas geniculata* (38.55%), followed by *S. pavanii* (21.72%), *Blautia coccoides* (4.81%), *S. retroflexus* (1.23%), *S. terrae* (0.56%), *S. maltophilia* (0.44%) and *Alkaliphilus crotonatoxidans* (0.38%). Noticeably, a significant percentage (30.68%) of species in DX100 was identified to be unknown.

### Microbial transformation of A- and B type trichothecene mycotoxins

**Fig. 2:**
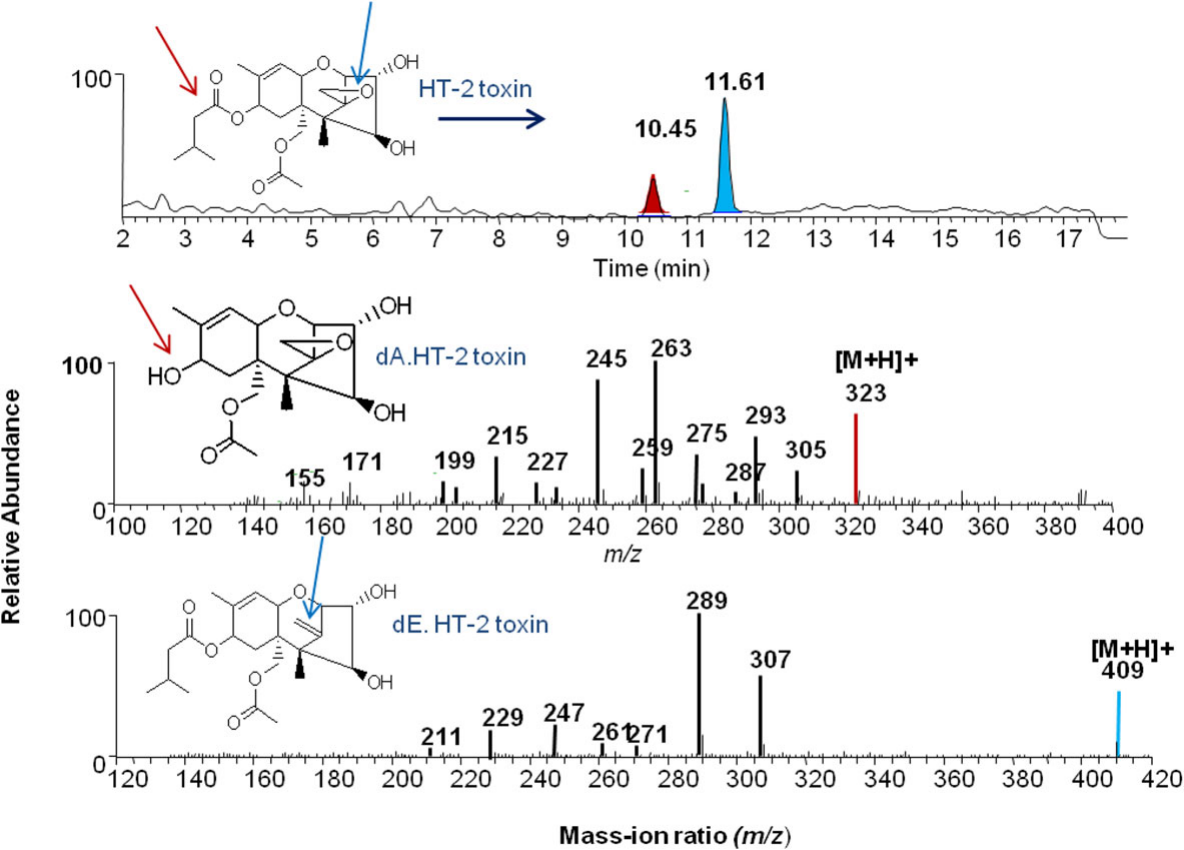
Mass spectrometric evidence of microbial de-epoxidation and de-acetylation of HT-2 trichothecene mycotoxin. The upper panel illustrates the mass spectrometer chromatograph of the de-acetylated and de-epoxidized products. The *middle* and *lower panels* show the mass spectra of the transformed compounds. *Arrows* on the *upper panel* indicate the targeted acetyl (*red*) and epoxy (*blue*) functional groups of HT-2 toxin. *Red* and *blue arrows* on the *middle* and *lower panels* identify the degradation sites of HT-2 toxin. dA.HT-2 = de-acetylated HT-2, and dE.HT-2 = de-epoxidized HT-2 toxin

Aerobic cultures of DX100 grown for 72 h in NB + 100 μg/mL HT-2 transformed 74% of the mycotoxin to de-epoxy HT-2 toxin, and 26% to a de-acetylated derivative (Fig. 2, Table 1). The culture incubated with T2-toxin transformed 46% of the mycotoxin to the de-epoxy T-2 product, 16% to an unknown compound and left 38% of the toxin untransformed. In the DX100 culture containing diacetoxyscirpenol (DAS), 61% and 39% was transformed into de-epoxy DAS and de-acetylated DAS, respectively. DX100 transformed 55% neosolaniol (NEO) to de-epoxy NEO and 45% to de-acetylated NEO derivatives. The bacterial culture showed capacity to transform T2-triol into two derivatives, namely: 47% de-epoxy T2-triol and 53% de-acetylated T2-triol.

**Table 1:** Transformation of type-A and B trichothecene mycotoxins by the DX100 microbial consortium. DAS = diacetoxyscirpenol, DON = deoxynivalenol, FUS = FusarenonX, 3-ADON = 3-acetyl-deoxynivalenol and 15-ADON = 15-acetyl-deoxynivalenol

Trichothecene mycotoxins		Untransformed trichothecenes (%)	Transformed products of trichothecenes (%)		
			De-epoxy	De-acetylate	Other^a^
Type-A	HT-2 toxin	0	74 ± 2.08	26 ± 2.08	-
	T-2 toxin	38	46 ± 0.58	-	16 ± 0.58 (unknown)
	DAS	0	61 ± 2.33	39 ± 2.33	-
	Neosolaniol	0	55 ± 2.51	45 ± 2.51	-
	T2-triol	0	47 ± 1.15	53 ± 1.15	-
Type-B	DON	0	100 ± 0.33	-	-
	Nivalenol	0	92 ± 3.38	-	8 ± 3.38 (DON)
	3-ADON	0	59 ± 1.76		41 ± 1.76 (DON)
	15-ADON	0	100 ± 0.66	-	-
	FUS	5	95 ± 2.90	-	-
	Varrucarol	3	97 ± 1.45	-	-

^a^Transformation of trichothecene mycotoxins to either DON or an unknown compound. The mean values and average ± standard errors of five replicate observations are presented

DX100 was also grown under aerobic conditions for 72 h in NB + 100 μg/mL of five type-B trichothecenes, separately. NIV was completely transformed into 92% de-epoxy and 8% DON products. The culture transformed 3-acetyldeoxynivalenol (3-ADON) into three products, namely: de-epoxy NIV (5%), de-epoxy DON (54%) and DON (41%). DX100 was able to transform acetyldeoxynivalenol (15-ADON) into de-epoxy 15-ADON (8%) and de-epoxy deoxynivalenol (dE-DON) (92%). Most (97%) of verrucarol (VER) was converted to de-epoxy VER, and 95% FusarenonX (FUS) was transformed into de-epoxy FUS. The remaining 3% VER and 5% FUS were not transformed. No spontaneous transformation of trichothecenes in the negative controls was observed.

### Effects of different growth factors on bacterial de-epoxidation activity

**Fig. 3:**
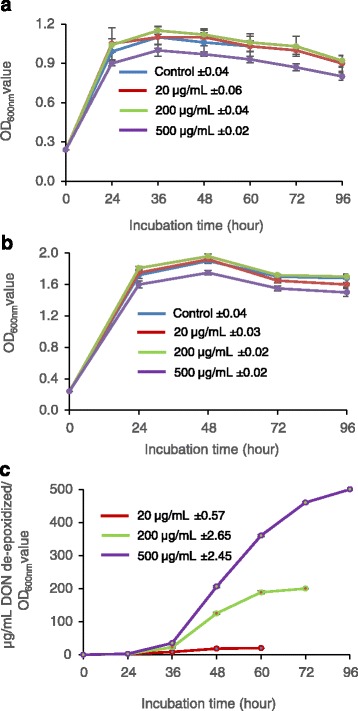
Effect of deoxynivalenol concentrations on the growth and de-epoxidation activity of DX100 microbial consortium. **a**: Microbial cell density (OD_600nm_) in MSB with different DON concentrations measured at 24, 36, 48, 60, 72 and 96 h. **b**: Microbial cell growth (OD_600nm_) in NB with different DON concentrations, measured at 24, 48, 72 and 96 h. **c**: Temporal DON de-epoxidation activity of DX100 in MSB containing 20, 200 and 500 μg/mL DON. The relative activity was measured by quantifying de-epoxidation activity divided by microbial growth (OD_600nm_ value) at different time points. Results are the mean of three replicate observations, and bars shown are ± standard errors of the means. The averages ± standard errors for three treatments and the control are presented in the graph. MSB = broth containing six mineral salts + 0.5% Bacto Peptone, DON = deoxynivalenol, and NB = nutrient broth

To determine if trichothecene concentration can affect the growth and de-epoxidation activity of DX100, the most common contaminant in food, namely DON, was tested. Growth of DX100 bacteria, aerobically, in MSB with 20 and 200 μg/mL DON was not significantly different from that observed in control cultures without the trichothecene (Fig. 3a). However, a reduced rate of bacterial growth was determined in MSB containing 500 μg/mL DON. Similarly, increasing the mycotoxin concentration up to 200 μg/mL did not affect bacterial growth in NB medium (Fig. 3b). Bacterial de-epoxidation activity [determined as μg/mL DON converted/cell density (OD_600_)] was lowest in MSB + 20 μg/mL DON, and highest in MSB + 500 μg/mL DON containing cultures (Fig. 3c). The highest rate of dE-DON formation was 11.5 μg/h in DX100 cultures in MSB with 500 μg/mL DON between 48 to 72 h. All of the mycotoxin was converted in the cultures containing 20 μg/mL between 48–60 h and in the cultures containing 200 μg/mL between 60–72 h. The consortium showed complete de-epoxidation of a high quantity of DON (500 μg/mL) within 96 h. In 200 μg/mL and 500 μg/mL of DON, the rate of de-epoxidation increased significantly after 36 h.

**Fig. 4:**
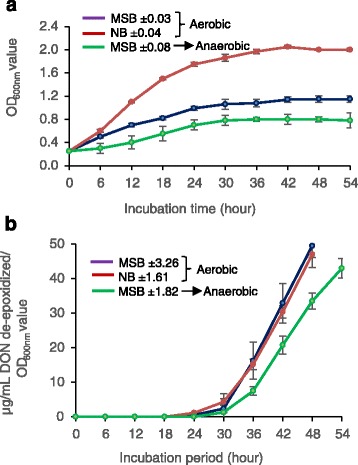
Relative growth and de-epoxidation capabilities of DX100 microbial consortium under aerobic and anaerobic conditions at moderate temperature. **a**: Microbial growth (determined by OD_600_ value) under aerobic conditions in MSB and NB medium and anaerobic conditions in MSB medium. **b**: Microbial DON de-epoxidation ability under aerobic conditions in MSB and NB media and anaerobic conditions in MSB medium. Tubes containing MSB or NB were supplemented with 50 μg/mL DON. The activity was measured by quantifying de-epoxidation activity divided by microbial growth (OD_600_ value) at different time points. Values are means of triplicate experiments, and bars shown are ± standard errors of the means. The average ± standard errors for the treatments are indicated in the graph. MSB = broth containing six mineral salts + 0.5% Bacto Peptone, NB = nutrient broth, DON = deoxynivalenol

DX100 grew more quickly in aerobic conditions in MSB + 50 μg/mL DON than in MSB + 50 μg/mL under anaerobic conditions (Fig. 4a). Aerobic growth was faster in broth containing NB + 50 μg/mL trichothecene compared to MSB + 50 μg/mL. However, the de-epoxidation activities, on a cell density basis, were very similar in NB + 50 μg/mL DON compared to MSB + 50 μg/mL (Fig. 4b). Also, de-epoxidation activity of DX100 under aerobic conditions in MSB + 50 μg/mL trichothecene was higher than in the same medium under anaerobic conditions.

**Fig. 5:**
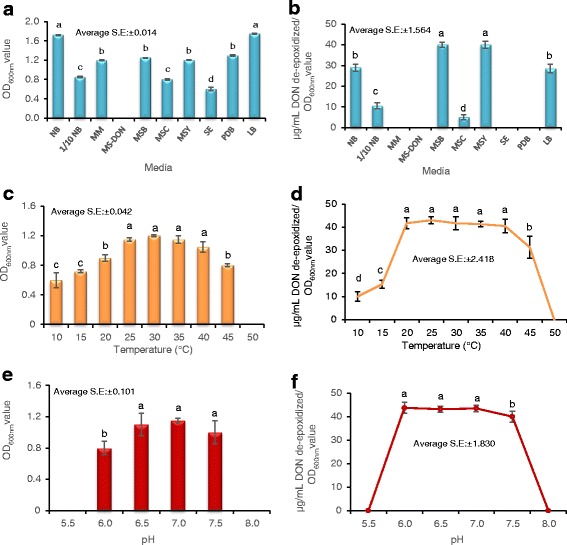
Effects of temperature, substrate and pH on growth and de-epoxidation activities of DX100 under aerobic conditions. **a**: OD_600nm_ values show relative growth performance of DX100 in different broth media. **b**: Bacterial de-epoxidation activity (DON to dE-DON transformation) in different broth media. **c**: OD_600nm_ values demonstrate the effects of temperatures on the growth capacity of DX100 in MSB. **d**: Bacterial DON de-epoxidation in μg/mL under different temperatures in MSB. **e**: OD_600nm_ values show growth behavior of DX100 at different pHs in MSB. **f**: Bacterial de-epoxidation (DON to dE-DON transformation) in μg/mL in different pHs. The activity was measured by quantifying de-epoxidation activity divided by microbial growth (OD_600_ value) under different growth conditions. Results are the mean of five replicate observations, and bars shown are ± standard errors of the means. The averages of ± standard errors for different growth conditions are presented in the graph. Treatments labeled with the same letter are not significantly different, according to Tukey’s HSD test (*p* < 0.05). Media: NB = nutrient broth, 1/NB = 1/10 strength NB, MM = Minimal medium, MS-DON = six mineral salts having 200 μg/mL DON. dE-DON = de-epoxy deoxynivalenol, MSB = six mineral salts + 0.5% Bacto Peptone, MSC = six mineral salts broth + 0.3% Bacto Peptone, MSY = six mineral salts with 0.5% yeast extract, S.E = standard error, SE = soil extract, PDB = potato dextrose broth and LB = Luria Bertani

Growth of DX100 in media containing 50 μg/mL DON under aerobic conditions at 72 h was three-fold higher in NB and LB than in SE and 1/10 NB and did not occur in MS-DON (Fig. 5a). Growth in MM, MSB and MSY was intermediate. Over the 72 h, de-epoxidation per cell density was highest in MSB and MSY and approximately 25% lower in NB and LB and lower by 75% and 87% in 1/10 NB and MSC, respectively (Fig. 5b). No de-epoxidation was observed in MM, MS-DON, SE and PDB in 72 h. Growth of DX100 under aerobic conditions for 72 h in MSB + 50 μg/mL DON was greatest between 25-40 °C, but no growth occurred at 50 °C (Fig. 5c). De-epoxidation activity was greatest and equivalent between 20-40 °C and lower at 10, 15 and 45 °C (*p* < 0.05) (Fig. 5d). Growth of the consortium under aerobic conditions for 72 h in MSB + 50 μg/mL DON was limited to pHs near neutral (i.e. 6.0 - 7.5) and the activity was not significantly different between pH 6.5 to 7.5 (*p* < 0.05) (Fig. 5e, f). No de-epoxidation occurred below 6.0 or above 7.5 this pH.

### Energy requirements and localization of bacterial de-epoxidation enzyme

Treatment of DX100 microbial consortium with the 0.001% and 0.01% NaN_3_ for 72 h reduced de-epoxidation activity to 5% (2.5 μg/mL) and 0%, respectively. In contrast, the control with no NaN_3_ added was unable to inhibit de-epoxidation activity of DX100. A cell lysate of DX100 transformed 20% (10 μg/mL) of 50 μg/mL DON to dE-DON within 72 h. In contrast, a cell-free culture filtrate did not show de-epoxidation activity, although the positive control having bacterial cells showed 100% de-epoxidation activity.

## Discussion

This study reports a highly enriched, stable and effective microbial consortium DX100 capable of de-epoxidizing eleven trichothecene mycotoxins under aerobic and anaerobic conditions and broad range of temperatures. Since co-applications of multiple microbial strains often have greater beneficial activity [22, 36, 37], the DX100 microbial consortium has a great potential to be used as an efficient biodetoxifier of animal feedstuffs contaminated with multiple co-contaminating trichothecene mycotoxins; therefore, improving food/feed quality and safety.

The microbial consortium was found to be comprised of over 30% of unknown bacterial species. This indicates the possibility of novel bacterial involvement in the trichothecenes transformation, and such bacteria appeared to be difficult to isolate using the currently available methods. Our attempts, over a period of a few years, failed to produce a stable pure strain with trichothecene de-epoxidation activity. Although a DON de-epoxidizing aerobic bacterium (*Citrobacter freundii* A47) was isolated from the same source as the DX100 [35]. Unfortunately, the strain did not retain DON de-epoxidizing activity after subculturing on agar media. Also the bacterium could not be detected in DX100, suggesting that the strain died during enrichment process. Isolation of trichothecene de-epoxidizing bacteria through selective enrichment procedures is also challenging because the de-epoxidation reaction involves removal of an oxygen molecule [31, 38], which does not supply any nutrients to bacteria [39, 40] preventing the utilization of trichothecenes as sole carbon or nitrogen sources for selective isolation of de-epoxidizing bacteria from soil samples. This is supported by the present work, which demonstrated that DX100 was unable to grow in media containing DON as a sole carbon source. Previous attempts to obtain aerobic bacteria with de-epoxidation activity were also successful, but the cultures lost their ability to detoxify trichothecenes after subculturing [29, 30]. Remarkably, DX100 consortium which was achieved through prolonged incubation of soil culture with high concentrations of DON demonstrated stable activity under aerobic conditions by de-epoxidizing trichothecenes after 100 generations in broth medium, and retained its activity when it was tested after two years of preservation at −80 °C.

Although trichothecenes are toxic to eukaryotes, prokaryotes appear to be resistant to these fungal secondary metabolites [30, 41]. In agreement with those findings the present study demonstrated that DX100 grew well in cultures with high concentration of DON (500 μg/mL) and metabolized it completely. Taking advantage of this capacity, we were able to increase the population of trichothecene de-epoxidizing bacteria in a culture by prolonged subculturing of soil microbes in media with 200 μg/mL DON. This is supported by the observation that a 50-fold dilution of DX100 showed de-epoxidation activity, while before the enrichment up to 20-fold dilution of the original culture (which used for enrichment in this study) was able to de-epoxidize trichothecene. This suggests that during each of twelve seven-day long enrichment steps the de-epoxidizing bacterial cells survived by gaining energy from co-metabolically cleaving the epoxy ring of the trichothecene, while the non-trichothecene metabolizing microbial populations declined by starvation. This explanation is supported by our observation that microbial de-epoxidation in broth mainly occurred at the stationary growth phase, when the bacteria had fewer nutrients, since at this growth stage bacteria increased their de-epoxidation rate when grown with higher concentrations of DON (500 μg/mL). Such microbial energy gain from co-metabolic reactions has been reported by Chiu and colleagues [42].

Treating the DX100 culture with 0.01% NaN_3_ completely blocked de-epoxidation activity. NaN_3_ inhibits cellular energy production, specifically in gram-negative bacteria, by interfering with catalase/cytochrome c oxidase activity, while gram-positive bacteria are mostly resistant to this energy inhibitor [34, 43]. This suggests a possible role for certain gram-negative bacteria in the observed trichothecene de-epoxidation activity [30]. Although the next generation metagenomics technology identified a low percentage of gram-positive anaerobic bacterial species, namely *Alkaliphilus crotonatoxidans*, *Bacillus* spp., and *Blautia coccoides*, the aerobic species of *Stenotrophomonas* (gram-negative) were found to be the predominant ones present in DX100. It remains unknown if the de-epoxidation of the variety of trichothecenes observed in the present study is the additive activities of a number of individual bacteria and/or the consequences of intra- and interspecific microbial interactions. However, interpretation of the data predicts that certain strains of *Stenotrophomonas* could be the major agents for aerobic de-epoxidation of trichothecenes, and the role of gram-positive bacteria (which are strictly anaerobic and endospore forming) could attributed to anaerobic de-epoxidation. Strains belonging to *Stenotrophomonas* demonstrated efficient biodegradations of fungal toxins and other environmental pollutants [44]. These bacteria were not identified as the most abundant ones in our initial soil-derived microbial culture that was enriched to obtain DX100. Therefore, the increase of de-epoxidation activity of DX100 could be further attributed to the elevation of de-epoxidizing bacterial population during the three-month enrichment process with high concentration DON.

Among 11 trichothecenes tested, DX100 efficiently transformed 10 to de-epoxy forms and/or DON and/or unknown compounds. Probably, because of the activity of specific microbial dehydrooxylase(s) NIV and 3-ADON were transformed to DON via removal of a hydroxyl group at the C4 position and the acetyl moiety, respectively. However, the reasons for DX100’s inability to subsequently de-epoxidize DON in those two assays are obscure.

Microbial de-epoxidation in this study occurred in intact and lysed bacterial cells. This implies that the small molecule DON entered into bacterial cells and became metabolized there by bacterial cytoplasmic enzymes. This agrees with previous studies that suggested that absorption of trichothecenes by human cells occurred via simple diffusion, and that bacterial cytoplasmic flavoproteins were involved in de-epoxidation of epoxyalkanes [45, 46]. The gene(s) and enzymes involved in trichothecene de-epoxidation have not been identified yet, however, the reductive nature of the bacterial trichothecene de-epoxidation reaction and inhibition of de-epoxidation activity by NaN_3_ suggests that an active electron transport process is essential and an epoxy reductase is involved in this biological reaction [30].

The functionality of such reductases appeared to be greatly affected by media composition [47, 48]. For example, this study showed that decreasing the concentration of Bacto Peptone in MS (from 0.5% to 0.3%) dramatically lowered the bacterial de-epoxidation performance, suggesting that the DON de-epoxidation activity of DX100 relies on the availability of certain proteins and amino acids. The inhibition of microbial de-epoxidation performance in high sugar containing MM and PDB media suggests the repressive effects of high sugar contents on the de-epoxidation enzymatic activity of DX100 [49]. Therefore, a screening strategy for microbial trichothecene de-epoxidation should use different media and enrichment procedures in parallel, as was done in the initial enrichment process of DX100.

All together, the achievement and characterization of DX100 microbial consortium may facilitate the development of an effective technology for biodetoxification of trichothecene contaminated foods and animal feeds. Furthermore, the knowledge we have gained from this study would assist in isolating the trichothecene de-epoxidizing genes and enzymes, which could, potentially, also be used to detoxify foods and feeds.

## Conclusions

The capacity of DX100 to de-epoxidize several mycotoxins could be an advantage to develop effective strategies for minimizing the synergistic/additive toxic effects of these toxic compounds on consumer health. The de-epoxidation activity of DX100 under aerobic and anaerobic conditions and wide range of temperatures as well as its ability to retain activity after many generations and years makes it a unique microbial source. Following similar formulation method to Mycofix® (encapsulation of bacteria) DX100 can be used as a feed additive [27] as well as the consortium has a potential to be utilized as a biodetoxifier of feedstuffs. Such applications of the consortium are pending on positive evaluation of the de-epoxidation performance of DX100 on contaminated feedstuffs followed by studying the suitability and safety of the treated materials for feed and food uses. Further improvement of de-epoxidation activity under nutrient rich conditions via increasing inoculum size and/or optimizing microbial growth factors could make the consortium an effective feed detoxifier. As an alternative, the trichothecene de-epoxidizing enzymes that might be obtained from DX100 through fermentation or a suitable aerobic process could be utilized for treatments of contaminated foods and feeds. Furthermore, analyses of meta-transcriptomes of active and inactive DX100 consortium could lead to the identification of novel genes responsible for aerobic de-epoxidation of trichothecenes. Since the fungal toxins enhance disease severity in grain [50], transfer and constitutive expression of the identified novel gene(s) into cereal genomes could generate cultivars resistant to *Fusarium* pathogens and their toxins. Such achievements not only improve yields and quality of grain but also minimize the exposure of humans and animals to these health hazardous fungal toxins while protecting the environment by reducing the applications of toxic chemicals in the environment for controlling trichothecene producing fungal pathogens.

## Abbreviations

ADON: 3-acetyldeoxynivalenol
DAS: Diacetoxyscirpenol
dE-DON: De-epoxy DON
DON: Deoxynivalenol
FUS: FusarenonX
LB: Luria bertani
LC-UV-MS: Liquid chromatography-ultraviolet-mass spectrometry
MM: Minimal medium
MSB: Mineral salts broth containing 0.5% Bacto Peptone
MSC: Six mineral salts broth containing 0.3% Bacto Peptone
MS-DON: Mineral salts broth containing DON
MSY: Six mineral salts broth containing 0.5% yeast extract
NaN_3_: Sodium azide
NB: Nutrient broth
NEO: Neosolaniol
NIV: Nivalenol
OD: Optical density
PDB: Potato dextrose broth
rpm: Revolution per minute
SE: Soil extract
VER: Verrucarol
